# 

**Published:** 1891-10-10

**Authors:** 


### NOTICE TO CORRESPONDENTS.

All MS., Letters, Books for Review, and other matters intended for the Editor should be addressed The Editor, The Lodge, Porchester Square, London, W.

The Editor cannot undertake to return rejected M.S., even when accompanied by stamped directed envelope.

**Notice to Advertisers and Subscribers.**

All Advertisements, Orders for Copies of The Hospital, and Business Communications should be addressed to The Publisher, *not to the Editor*, The Hospital, 140, Strand, W.C.

The Cost of Subscription, post free, is as follows:—Per week, 1½d.; per quarter, 1s. 8d.; per half-year, 3s. 3d.; per annum, 6s. 6d. Foreign:—Per annum, 8s. 8d.; payable, in all cases, in advance.

**NOTICE.—The Index for Vol. IX., (ending March, 1891) is on sale at the Office of this Journal, price 2½d., post free.**

### General Charities.

[This Column is devoted to an account of work of charitable institutions other than Medical Charities. The Editor will be glad to receive reports or news of work at 140, Strand. *Philanthropy* should be plainly written on the envelope.]

A special benefit has been arranged at the Royal Aquarium, Westminster, to take place on November 3rd next, on behalf of the **Gordon Boys' Home** at Chobham, near Woking, and in furtherance of the appeal recently made for £40,000 by Lord Tennyson.

A new home for twenty-five boys was opened last week in connection with **The Swanley Orphan Homes**, in which there are at present about 180 boys. The new home has been given by Mrs. Morrison, of Horton Kirby, Kent, in memory of her late husband. As Lord Herschell, who presided at the opening, pointed out, it has been found possible here to combine the ordinary elementary education with industrial training and discipline, and to do that without in the least impairing the efficiency of the ordinary elementary education. The problem which has thus been solved in so satisfactory a manner is one of difficulty and of great importance. It appears that the income of the Institution is at present much below the expenditure, and Lord Herschell made a strong appeal on behalf of the efforts which the Committee are now making to raise the £5,000 which they require to meet the immediate needs of the homes.

The first law and regulation of the institution called **The London Aged Christian Society** is, that its objects shall be the permanent relief of the decidedly Christian poor of either sex who have attained the age of sixty-five years, and who reside within five miles of St. Paul's Cathedral. Previously to receiving the Society's report we were under the impression that people are either Christians or not Christians, and were not aware of the existence of the superior being termed the "decidedly Christian"—whatever that may mean. Presumably the Committee of Management take upon their shoulders the invidious task of settling as to who are "decidedly Christian," but it would be interesting to know what standard they go by, and how they can possibly decide such a knotty question. During the past year the Society, whose income amounted to £1,506 1s. 2d., paid the sum of £887 2s. to their very superior body of pensioners, so that they are in the happy position of finding it unnecessary to appeal to the public for assistance.

The Hon. Secretary to the **St. James' Home for Inebriates**, Kennington Park, appeals for the sum of £400 to pay off a pressing debt, and rent, and rates. The Home was started fifteen years ago for the purpose of rescuing women and girls from the thraldom of strong drink, which unfits them totally, not only for the duties of wives, mothers, and daughters, but for the ordinary work of life. There is a generally prevailing idea that it is impossible to reclaim drunken women, and the institution was started some fifteen years ago with a view to refute this idea. The Hon. Secretary complains that the funds of the Society are drained, but we are constrained to ask whether this unsatisfactory state of affairs would have come to pass if the management had confined their attention to the strict work of the fund. It appears, upon their own admission, that they accepted during the past winter the responsibility of providing meals to the number of 159,525 for hungry crowds who besieged them during the severe weather. It is this which they allege caused them to fall into debt, and we fail to see how they were justified in expending money in this direction. The Society was founded and exists for the purpose of providing a home for female inebriates, and no mention is made in their rules of any further scope.

Among the various wants of **The Royal Female Philanthropic Society** are new members for the working guild of help, and more visitors to the home at Manor Hall, Great Church Lane, Hammersmith, who would interest themselves in individual cases suggested by the Committee, or occasionally read to the girls while at needlework. The Society was established by the late Miss Neave, a fellow-worker with Mrs. Fry, who saw how greatly girls discharged from gaol stand in need of a home in which they can be trained to start afresh in life with some reasonable hope of earning an honest living. The class of girls which enters the home is not an easy one to deal with; habits of idleness of long standing, absence of self-control, ignorance, dishonesty, and untruthfulness are weeds hard to destroy. Such tendencies to evil can with difficulty be counteracted during a stay of two years in the home, which is the longest period allowed for influencing the inmates. We presume that narrowness of means has prevented the management from giving a longer training to the young women, who are instructed in every branch of household work. The age of admission is fifteen years and upwards, and parents or friends of those admitted are required to pay 4s. per week towards their support. Suitable clothing must be provided on entrance, but afterwards the inmates are clothed by the institution, and those who do well receive an outfit for service on leaving. Cases of immorality, for which female penitentiaries are provided, are inadmissible; those only being eligible who have been convicted and imprisoned for a first offence, discharged from service for dishonesty, but not prosecuted, and those who are destitute through the vice or loss of parents.

### Scraps and Gleanings.

The General House to House collection at Grantham, in aid of the Hospital, has resulted in contributions to the amount of £133.

The decoration of the Leicester Children's Hospital is completed. The institution now claims being the most beautiful of its kind in England.

Last week a demonstration, in aid of the St. Albans Hospital, was organised by the Foresters of Park Street and Frogmore. £18 was collected.

The Committee of the Sanitary and Infectious Diseases Hospital at Devizes have decided to expend £400 on two additional wards for the Institution.

At a meeting of the Corporation of London yesterday, a sum of 200 guineas was voted to the relief of the sufferers by the Spanish inundations.

The transference of the Bedford Small-pox Hospital from the Board of Guardians to the Rural Sanitary Authorities is now under consideration.

The Bristol Royal Infirmary is in a bad way. A debt to the extent of £5,000 is anticipated by the end of the year. Legacies and subscriptions both show a falling off.

A grateful patient of the Royal Ear Hospital, Soho Square, has offered to bequeath himself as a legacy to the hospital for purposes of scientific investigation.

The Sanitary Institute, 74, Margaret Street, Regent Street, advertise a course of lectures for candidates for sanitary offices. A fee of £1 1s. for the course of twelve lectures is charged.

The annual church parade of friendly societies in connection with the Devizes Cottage Hospital took place on Sunday last, and proved very successful. The collections show an increase over last year.

A favourable report was read at the annual meeting of the Lowestoft Hospital. The accounts show a balance in favour of the institution. The number of patients treated has exceeded that of previous years.

Although the scheme for a joint hospital for the Dewsbury Union has been so long under consideration, the authorities have not yet ascertained which districts will be willing to join, or which decline to do so.

At the North-Western Poor Law Conference the Right Hon. J. Hibbert, the President, stated that Norfolk contains the largest number of paupers receiving relief of the English counties, and Lancashire the fewest.

On Sunday an open-air demonstration got up by the Friendly Societies in the district, was held at Brampton, the object being the support of the Chesterfield and North Derbyshire Hospital. The total result of the collections was £10 4s.

One of the leading branches of the Prisoners' Aid Society, has volunteered to make a grant of 5s. per week towards the maintenance and expenses of finding work for any of their cases that are received into the Church Army Labour Home.

The Bishop of Durham laid the foundation-stone of the new isolation ward at the Sunderland Infirmary on the 28th ult. Miss Forster, of South Hetton Colliery, is bearing the expense of the building, which will amount to upwards of £2,700.

At the annual meeting of the Victoria Hospital for Sick Children, Hull, two new wards were named. These were opened earlier in the year by the Marchioness of Salisbury. A portrait of the late Sheriff Allison was presented and unveiled.

An appeal for a central hospital has just been made at Ebbw Vale, Wales. At the meeting at which the proposal was made it transpired that the present existing hospital had received eight patients during the eight years it had been opened.

The Society for Home Teaching for the Blind, 31, New Bridge Street, E.C., does much to render the lives of the blind more bearable. Free teachers are sent on application to give instruction to those who desire to learn Moon's type. Funds are greatly needed.

The Hove Hospital and Dispensary are justly to be congratulated. A generous unknown friend has shown an appreciation of the Institution, which it fully merits, by a gift of £1,000. Three thousand in and out patients have received treatment since January last.

The Cork Dispensary Committee have been busy quarrelling over the election of a doctor for a considerable time. Now that they have time to turn to business, they will find plenty to do remedying defective disinfection cases, relating to which neglect is very apparent.

The occasion of the annual hospital demonstration at Middlesborough was seized by Mr. Tom Mann to demonstrate to the meeting that excessive work was one means of filling the hospital. He stated that at Middlesborough the blast furnacemen work 84 hours a week, which was a disgraceful fact.

A sale of work was held in the Hull Royal Infirmary on September 24th and 25th, on behalf of the Samaritan Fund. The stalls were held by nurses, who have been zealous workers for the sale. Decorations were lent, and refreshments given, by various tradesmen and friends. The sale realized £80.

St. Margeret's Catholic Industrial School have been fortunate in passing on a refractory scholar to the Croydon Industrial School. The girl absconded, and then spread prejudicial reports concerning the school. These reports were not substantiated, and the managers are well rid of a tiresome inmate.

The *Post* quotes a letter from the Hon. Samuel Parker, Minister for Foreign Affairs at the Hawaiian Islands, in reference to the monument to be erected in memory of the late Father Damien at Molokai at the expense of the Leprosy Fund. The monument will be erected on the selected site without cost to English friends.

The memorial stone of the extensive block of buildings in course of erection for the infirmary of the Romford Union was laid last week. The buildings will consist of a centre block of two floors, and will have a wing on each side. Each floor will hold twenty-eight beds, a duty room, nurses' room, &c. The whole will be built on the pavilion principle, and will be connected by a corridor. The new works have been designed by Mr. F. Whitmore, architect, Chelmsford.

### THE STUDENT OF MEDICINE.

[All communications for this Supplement should be addressed to THE EDITOR of THE HOSPITAL, at 140, Strand, with the words, "Students' Column," written in the left hand top corner of the envelope.]

### APPOINTMENTS.

At the Middlesex Hospital, George D. B. Levick, M.R.C.S., L.R.C.P., L.S.A., has been appointed house surgeon. At the West London Hospital, Hammersmith Road, Frank Belben, M.B., B.C., Cantab., has been appointed house physician; and George F. Murrell, M.R.C.S., L.R.C.P., house surgeon. At the Poplar Hospital for Accidents, E. Robert D. Muir, M.R.C.S., L.R.C.P., has been appointed assistant house surgeon; and Ernest G. H. Williams, M.R.C.S., L.R.C.P., senior house surgeon. At the Halifax Infirmary and Dispensary, John A. Adams, M.A., M.B., has been appointed assistant house surgeon. At the Salford Royal Hospital, Robert Ellis Lord, M.B. and B.Sc.Lond., M.B., Ch.B. Vict., M.R.C.S. Eng., has been appointed senior house surgeon; and Charles Christopher Heywood, M.B. and B.C., M.A.Camb., M.R.C.S., L.R.C.P., junior house surgeon. At the Parish of St. Leonard, Shoreditch, F. L. P. Sawell, M.R.C.S.Eng., L.S.A., has been appointed resident assistant medical officer. At the Stourbridge Dispensary, John S. Maynard, M.B., and C.M. Edin., has been appointed house surgeon and secretary.

### VACANCIES.

The following vacancies are announced this week, the amount of salary in each case being given after the name of the institution:

**Resident Medical Officers.**

Cheltenham General Hospital, House Surgeon—Salary £80, &c.

Cheshire County Asylum, Upton, near Chester, Junior Assistant Medical Officer—Salary £120, &c.

City of London Hospital for Diseases of the Chest, Victoria Park, E., House Physician—Board, &c.

Kent County Asylum, Barming Heath, Maidstone, Third Assistant Medical Officer—Salary £150, &c. Also Fourth Assistant Medical Officer and Pathologist—Salary £150, &c.

Paddington Green Children's Hospital, W., House Surgeon—Salary £0, &c.

Royal Isle of Wight Infirmary and County Hospital, Ryde, House Surgeon and Secretary—Salary £60, &c.

**Non-Resident Medical Officers.**

Dental Hospital of London, Leicester Square, Anæsthetist.

Hampstead Provident Dispensary, Medical Officer.

### Charing Cross Hospital Medical School.

The Entrance Scholarship, value 100 guineas, has been awarded to Mr. J. R. Langley, and that of 50 guineas to Mr. Howard Green.

### SCHOLARSHIPS AND PRIZES.

### University of Aberdeen, Summer Session, 1891.

CLASS OF PRACTICAL MATERIA MEDICA AND PHARMACY.

MEDALS AND FIRST CLASS CERTIFICATES.—J. Gillespie, Aberdeen, 85; A. Brown, M.A., B.Sc., Ordiquhill, and W. Hector, Bourtie, 82 5.

FIRST CLASS CERTIFICATES.—A. W. Mackintosh, M.A., Manse of Deskford, Banffshire, 80; R. Burgess, Wigton, 79; E. M Payne, Birmingham, 78·5; C. K. Morgan, Inverkeilor, Forfarshire, 77·5; F. A. Gill, Wick, and A. L. Cruickshank, M.A., Fraserburgh—equal, 75.

SECOND CLASS CERTIFICATES.—A. Duncan, Botriphnie, 71; G. A. Allwood, Jamaica, and J. Matheson, M.A., Plockton—equal, 70; P. Howie, Aberdeen, 69·5; D. Grant, Dores Parish, Inverness-shire, 69; J. W. Attegalle, Tangalle, Ceylon, 68·5; G. Marr, Bourtie, 68; J. Ingram, Clatt, 67·5; C. J. Milne, Agra, India, 66 5; W. C. Pieris, Colombo Ceylon, and R. A. Slater, Preston—equal, 65; A. Henderson, M.A., Aberdeen, 64; R. Mitchell, Fyvie, and A. A. Moore, Portsmouth—equal 63·5; J. F. Hall, Ettrick, 62·5; A.B.Cruickshank, Aberdeen, A. Ogston, M.A., Crathie, and C. W. Profeit, Tarland—equal, 60.

PRACTICAL PHYSIOLOGY.

MEDALLISTS.—J. Gillespie, Aberdeen, 89; A. Brown, M.A., B.Sc., Ordiquhill, Banffshire, 83, P. Howie, Aberdeen, 81.

FIRST CLASS CERTIFICATES.—H. M. Mitchell, Castledearg, Tyrone, Ireland, 76; D. Finlayson, Tain, Ross-shire, 75.

SECOND CLASS CERTIFICATES.—J. R. Kennedy, Tain, Ross-shire, 72; A. Ogston, M.A, Aberdeen, and M Polson, Aberdeen—equal 70; H. J. Dawson, Williamstone, Insch, 68; Hugh Fraser, Aberdeen, and A. W. Macintosh, M.A., Deskford, Cullen—equal, 67; P. M. Muttukumaru, Colombo, Ceylon, 66; Robert Burgess, Aberdeen, 64; J. Matheson, M.A., Stromeferry, Ross-shire, 61; Adam Alexander, Fyvie; J. F. Hall, Aberdeen, and George Sturrock, Aberdeen—equal, 60.

FOR THE BEST COLLECTION OF HISTOLOGICAL SPECIMENS—(MEDALLISTS).—Alexander Brown, M.A., B.Sc., Ordiquhill, Banffshire; George Sturrock, Aberdeen.

SUBSEQUENT ORDER.—A. Henderson, M.A, Aberdeen; C. K. Morgan, Arbroath; A. Alexander, Fyvie; R. G. M'Gowan, Norwich; J. Falconer Hall, Aberdeen; F. Ainley, Huddersfield; and C. W. Profeit, Balmoral.

